# Individual and contextual factors associated with childhood malnutrition: a multilevel analysis of the double burden of childhood malnutrition in 27 countries

**DOI:** 10.1186/s41256-022-00276-w

**Published:** 2022-11-24

**Authors:** Mukhtar A. Ijaiya, Seun Anjorin, Olalekan A. Uthman

**Affiliations:** 1 Jhpiego, Plot 971, Rueben Okoya Crescent, Off Okonjo Iweala Street, Wuye District, Abuja, FCT Nigeria; 2 Division of Health Sciences, Warwick Centre for Global Health, Warwick Medical School, University of Warwick, Coventry, CV4 7AL UK

**Keywords:** Multilevel, Malnutrition, Childhood

## Abstract

**Background:**

Malnutrition is a key global health challenge and a major contributor to childhood morbidity and mortality. In recent times, the contrasting coexistence of undernutrition including micronutrient deficiencies and overweight/obesity called double burden of malnutrition has been noted at individual, household or population level and/or at different times in life. The objective of this study was to examine individual, neighborhood and country level factors that are associated with the double burden of childhood malnutrition.

**Methods:**

We conducted multivariable multilevel logistic regression analyses on the most recent demographic and health datasets from surveys conducted between 2015 and 2020 in low- and middle-income countries. We analyzed data of 138,782 children (level 1) living in 13,788 communities (level 2) from 27 countries (level 3).

**Results:**

The results of our analysis show variation in childhood malnutrition across the 27 countries from as low as 6.5% in Burundi to as high as 29.5% in Timor Leste. After adjusting for all level factors, we found that those who were wasted/overweight tended to have had an episode of diarrhea or fever in the last two weeks preceding the survey, were part of a multiple birth, were being breastfed at the time of the survey and born to mothers with more than one under 5-child resident in neighborhoods with high illiteracy and unemployment rates. The intra-neighbourhood and intra-country correlation coefficients were estimated using the intercept component variance; 44.3% and 21.0% of variance in odds of double burden of childhood malnutrition are consequent upon neighborhood and country level factors respectively.

**Conclusions:**

Evidence of geographical clustering in childhood malnutrition at community and country levels was found in our study with variability due to neighborhood level factors twice that of country level factors. Therefore, strategies in tackling the double burden of malnutrition must consider these shared drivers, contextual barriers and geographical clustering effects.

**Supplementary Information:**

The online version contains supplementary material available at 10.1186/s41256-022-00276-w.

## Background

Malnutrition is a key global health challenge, it’s defined by the World Health Organization (WHO) as undernutrition which encompasses wasting, stunting and underweight; Micronutrient-related malnutrition; and overweight/Obesity [1]. Malnutrition is a major contributor to childhood morbidity and mortality, it predisposes children to various illnesses ranging from kwashiorkor to diet-related non-communicable diseases, reduction in productivity during the adult years and on a larger scale resulting into intergenerational poverty [2, 3]. Childhood malnutrition severely affect a child’s cognitive and physical growth and decreases adult Quality of Life (QoL).

Globally, an estimated 22% (149.2 million) of children under-5 were reported to be stunted, and a further 6.7% (45.4 million) of children under-5 were wasted in 2020 [3]. In addition, over 38.9 million (5.7%) of under-5 children were reported overweight [3]. Africa and Asia jointly account for 94%, 97% and 75% of all stunted, wasted and overweight children respectively [3]. At regional levels, every other region has seen a decline in the number of stunted children except Africa, although Southern Asia has the highest prevalence of stunting worldwide [3]. Similarly, Asia accounts for about 70% of all children affected by wasting, with southern Asia sub region accounting for about 50% of all children affected by wasting [3]. The number of overweight children have not seen any appreciable change in numbers and percentages in the last two decades, except in South Eastern Asia and Northern Africa who have seen significant increments [3].

In recent times, with the changes in dietary patterns and consumption; population disease burden; and demographic structure and life expectancy; undernutrition including micronutrient deficiencies now coexist alongside overweight/obesity [4, 5]. This contrasting coexistence is referred to as the double burden of malnutrition. This double burden happens at individual, household or population level and/or at different times in life [4, 5]. At individual level, this may involve the simultaneous occurrence of different forms of malnutrition at different life periods [4, 5]. Double burden households have multiple forms of malnutrition among household members at the same time, similar to existence at population level which denotes prevalence of multiple forms at specific geographical areas [4, 5]. Therefore, interventions to address malnutrition must tackle the shared drivers of the double burden.

The major contributors of double burden of malnutrition have been categorized into three underlying factors: social and demographic; biological; environmental and behavioral [4, 6]. In addition, multiple studies have highlighted factors associated with childhood malnutrition such as employment, poverty level, food security, conflict, parental education, government policies, and socio-economic inequalities [7–11]. Other factors such as recurrent illness, feeding problems and / or chronic diseases have been found to increase the risk of malnutrition in children [6, 12]. Beyond being highlighted as a cause of malnutrition, poverty with its attendant effect and interrelationships with the aforementioned factors, has also been identified as a consequence of the double burden of malnutrition [13]. This potentiating interconnectedness has been described as a vicious cycle [13]. In recent times, climate change has been a key factor in childhood malnutrition with its primary effect on food availability, access and utilization; and secondary effect on water, sanitation and exposure to health risks and diseases [6].

Child growth is a standard indicator for measuring nutritional status. This is done using anthropometric measurement to ascertain changes in body composition. Amongst children under 5 years old, the gold standard indicators to determine malnutrition are stunting- child being too short for his or her age; wasting- child being too thin for his or her height; underweight- child being too thin for his or her age; and overweight- child being too heavy for his or her height. The stunting indicator measures a child’s height and his/her age compared against less than 2 Standard Deviation (SD) of the WHO child growth standards median, while the underweight indicator is estimated through the measurement of a child’s weight and age compared against less than 2 SD of the WHO Child growth standards median [12, 14, 15]. Wasting is calculated by measuring a child’s weight and height in comparison to the WHO Child growth standards median same as the overweight indicator, with the former less than 2 SD of the WHO Child growth standards median and the latter greater than 2 SD of the median [14, 15]. Although these indicators use different metrics and make for different categories, they are not mutually exclusive.

Designing novel and integrated interventions which addresses the double burden of malnutrition requires an understanding of the shared drivers and contributors. We found significant number of literatures on the factors associated with wasting, undernutrition, stunting and overweight, however we found no extensive literature on the individual and contextual factors associated with the double burden of childhood malnutrition across countries. Multilevel regression analysis represents an ideal method of determining the shared factors and contributors associated with the double burden of malnutrition, their differences across levels and the magnitude of the clustering effects at these levels as well as cross level interactions [16]. From the foregoing, the objective of this study was to examine individual, neighborhood and country level factors that are associated with the double burden of childhood malnutrition.

## Methods

### Study design and data sources

This multilevel analysis was conducted using data from Demographic and Health Surveys (DHS) implemented by ICF International. These nationally representative cross-sectional household surveys provide data on maternal and child health, child survival, HIV/AIDS and other sexually transmitted infections (STIs), reproductive health and a whole range of healthcare areas, and are usually conducted every five years in each country. These surveys have been conducted in over 90 LMIC countries [17]. The surveys use a stratified multistage cluster sample design to collect data of women and men who are between age 15 and 49 years and their young children under five years of age living in randomly selected households which serves as the primary sampling unit [17]. DHS surveys use a minimum of two primary questionnaire types during data collection: Household Questionnaire, and Woman’s Questionnaire [17]. The child recode component of the most recent DHS surveys of twenty seven countries were included in this analysis. Only DHS datasets of countries which had the full complement of our required variables were included in this analysis.

### Variables

The double burden of malnutrition is defined by the coexistence of undernutrition and overweight and obesity, as well as diet-related noncommunicable diseases, within individuals, households, and populations, as well as across the lifecourse. In this study, we defined double burden of malnutrition at individual level. The outcome variable was derived from the WHO child growth standard Weight-for-Height [15]. The Weight and Height standard was derived from Weight and Length measurements expressed as Z-scores. Children with z-scores below 2 standard deviations are classified as moderately and severely wasted, while those with z-scores above 2 standard deviations are considered overweight, and those in between the aforementioned standard deviations are considered not malnourished [12]. Consequently, we defined the outcome variable as a factor variable with two categories: Not Wasted/Overweight and Wasted/Overweight. We chose weight for height as the anthropometric indicator because of its comparative robustness compared to the other indicators [12].

We included both child and maternal individual level factors as control variables. The child individual level factors include age, sex, child birth type, diarrhea in the last two weeks preceding the survey, fever in the last two weeks preceding the survey and breastfeeding status. The maternal individual level factors include age, education, marital status, employment status, number of under five children, wealth index as constructed by the DHS and maternal health behavior. The maternal health behavior was constructed with principal component analysis of these three individual level factors: mother’s knowledge of Oral Rehydration Salts (ORS), place of delivery and entries in the immunization roster.

Neighborhoods are defined as clusters of respondents in proximal households which serves as the primary sampling unit within the DHS dataset. These neighborhoods in the DHS datasets are stratified and selected with probability proportional to size, with households systematically sampled making it most suitable for intra and inter community analysis. The community level factors chosen include poverty level, neighborhood rurality, illiteracy and unemployment rates. Also included are unsafe sanitation, unimproved water source and limited access to health facility levels. All these were categorized into low or high with median value serving as the reference for the low and high groups.

The country level variables included in our model were Human Development Index (HDI), Total Fertility rate (TFR) and Cereal Yield (kg per hectare) obtained from the Human Development Report and the World Bank Databank respectively [18, 19]. The HDI which is a composite measure of life expectancy, education and income per capita was classified based on HDI fixed cut off points into low, medium and high according to the Human Development Report [19]. The TFR is the number of children that would be born to a woman at the end of their reproductive period subject to age-specific fertility rates [18]. The TFR was classified into low, ideal and high following the recommendations of the World Fertility Report [20]. The cereal yield (kg per hectare) categorized into three tertiles: low, medium and high; represents production data of cereal harvested for dry grain measured in kilograms per hectare of land [18].

### Statistical analyses

WE conducted descriptive, univariable analysis of the variables with respondents’ distribution expressed as absolute number (percentages) and mean (SD) for categorical and continuous variables respectively. The analysis was adjusted for sample weight, stratification, and clustering.

Multivariate multilevel logistic regression was used to analyze the individual, community and country level factors associated with childhood malnutrition of baby-mother pairs (level 1) living in a neighborhood (level 2) within a country (level 3). Five models were constructed: an empty model with no explanatory variable to determine the variance in odds of malnourishment between neighborhoods and countries. Model 2, 3 and 4 included individual-level, neighborhood-level and country-level variables only respectively. Model 5 included all individual-level, neighborhood-level and country-level variables.

Odds ratio at 95% credible interval (CrI) was used to report the associations between the variables (fixed effects). Variance (random effects) was measured by intra-class correlation (ICC) and median odds ratio (MOR). ICC assesses the similarity between respondents in the same neighborhood and in the same country [21, 22]. MOR estimates the variance expressed as odds ratio attributed to neighborhood and country contexts, and the variance is directly proportional to increasing MOR [23]. An MOR of one is an indication of no neighborhood or country variance [23].

Descriptive analyses were performed using STATA 17, and multilevel models were fitted using the MLwinN programme, version 3.05 in STATA17 using the Bayesian Markov Chain Monte Carlo procedure which yields unbiased estimates with better properties [24–26]. We employed Bayesian Deviance Information Criterion to assess model fit [27].

## Results

### Descriptions of survey data

We analyzed data of 138,782 children (level 1) living in 13,788 communities (level 2) from 27 countries (level 3) from DHS surveys that were conducted between 2015 and 2020. Across countries, the number of children ranged from 1082 in South Africa to 12,033 in Benin. The median number of neighborhoods was 466, with Senegal (214) and Nigeria (1378) having the least and the greatest number of neighborhoods respectively. Two thirds of the countries (18 of 27 countries) were in the Africa region and 22% (6 countries) from the Asia region. Americas, Europe and Oceania each had one country in the pooled dataset. More than half (15 of 27 countries) of the countries had low HDI, with 21 countries having a high TFR. With respect to cereal yield (Kg per hectare), 11 countries each were in the low and high categories respectively. Table 1 shows the countries, survey characteristics and key country level variables.

**Table 1:** Description of demographic and health surveys data by countries

Continent	Country	Survey year	Number of children	Number of neighbourhoods	Malnourished (%)	Human development index (HDI)	Total fertility rate (TFR)	Cereal yield (kg per hectare)
Europe	Albania	2018	2462	631	18.3	High HDI	Low TFR	Third tertile (highest)
Asia	Armenia	2016	1561	304	17.7	High HDI	Low TFR	Third tertile (highest)
Africa	Angola	2016	6407	625	8.6	Medium HDI	High TFR	First tertile (lowest)
Africa	Burundi	2017	6052	554	6.5	Low HDI	High TFR	First tertile (lowest)
Africa	Benin	2018	12,033	555	7	Low HDI	High TFR	First tertile (Lowest)
Africa	Cameroon	2019	4477	428	15.5	Medium HDI	High TFR	Second tertile
Africa	Gambia	2020	3811	279	7.5	Low HDI	High TFR	First tertile (lowest)
Africa	Guinea	2018	3430	399	15.1	Low HDI	High TFR	First tertile (lowest)
Americas	Haiti	2017	5598	449	7.4	Low HDI	Ideal TFR	First tertile (lowest)
Africa	Liberia	2020	2457	324	8.2	Low HDI	High TFR	First tertile (lowest)
Africa	Mali	2018	8588	345	10.9	Low HDI	High TFR	Second tertile
Asia	Maldives	2017	2362	260	13.3	High HDI	Low TFR	Third Tertile (highest)
Africa	Malawi	2016	5178	850	7.3	Low HDI	High TFR	Second tertile
Africa	Nigeria	2018	11,405	1378	9	Low HDI	High TFR	Second tertile
Asia	Nepal	2016	2369	375	11	Medium HDI	Low TFR	Third tertile (highest)
Oceania	Papua New Guinea	2018	3290	674	18.2	Low HDI	High TFR	Third tertile (highest)
Asia	Pakistan	2018	4151	554	9.5	Medium HDI	High TFR	Third tertile (highest)
Africa	Rwanda	2020	3809	500	6.9	Low HDI	High TFR	First tertile (lowest)
Africa	Sierra Leone	2019	4144	564	10.5	Low HDI	High TFR	First tertile (lowest)
Africa	Senegal	2019	5531	214	10.4	Low HDI	High TFR	First tertile (lowest)
Asia	Tajikistan	2017	5867	366	8.8	Medium HDI	High TFR	Third tertile (highest)
Asia	Timor-Leste	2016	5718	455	29.5	Medium HDI	High TFR	Third tertile (highest)
Africa	Tanzania	2016	8962	607	8.5	Low HDI	High TFR	Second tertile
Africa	Uganda	2016	4413	688	7.8	Low HDI	High TFR	Third tertile (highest)
Africa	South Africa	2016	1082	466	16.2	High HDI	Ideal TFR	Third tertile (highest)
Africa	Zambia	2019	8711	545	9.6	Medium HDI	High TFR	Third tertile (highest)
Africa	Zimbabwe	2015	4914	399	9.4	Medium HDI	High TFR	First tertile (lowest)

The mean age of the children included in this study was 28 months, and males constituted half (50.6%) of the total study population (Table 2). Only a tenth (10.5%) of the children were wasted/overweight, and this varied from 6.5% in Burundi to 29.5% in Timor Leste. Most of the mothers were married (90.0%), were between the ages of 25–34 years (50.2%) and in employment (58.7%). The community poverty level among the study mother children-pairs was 45.6%, similar to the community illiteracy level (43.5%), and community unemployment level (46.8%). Conversely, the neighborhood rurality rate was just 3.0%. Our descriptive findings show the majority of the children were resident in countries with low HDI (64.2%), low cereal yield (Kg per Hectare) (41.6%) and with high total fertility rate (89.4%).

**Table 2:** Summary of the 27 countries pooled sample characteristics of DHS data

Variables	Overall	Not wasted and overweight	Wasted and overweight	*P* value
Total		122,316 (89.5)	14,390 (10.5)	
Age in months mean (SD)	28.4 (17.3)	29.0 (17.1)	23.1 (17.5)	< 0.001
Sex				< 0.001
Male	69,116 (50.6)	61,219 (50.1)	7898 (54.9)	
Female	67,589 (49.4)	61,097 (50.0)	6492 (45.1)	
Child birth type				0.072
Single birth	132,734 (97.1)	118,807 (97.1)	13,927 (96.8)	
Multiple births	3972 (2.9)	3509 (2.9)	463 (3.2)	
Diarrhea in the last 2 weeks preceding the survey				< 0.001
No diarrhea	116,081 (85.2)	104,090 (85.4)	11,991 (83.6)	
Had diarrhea	20,213 (14.8)	17,868 (14.7)	2345 (16.4)	
Child fever in the last 2 weeks preceding the survey				0.473
No fever	107,760 (79.0)	96,467 (79.1)	11,294 (78.7)	
Had fever	28,623 (21.0)	25,570 (21.0)	3052 (21.3)	
Currently breastfeeding				< 0.001
No	84,532 (63.0)	77,523 (64.5)	7009 (49.9)	
Yes	49,715 (37.0)	42,674 (35.5)	7041 (50.1)	
Maternal age (Y)				< 0.001
15–24 years	36,601 (26.8)	32,497 (26.6)	4104 (28.5)	
25–34 years	68,629 (50.2)	61,465 (50.3)	7163 (49.8)	
35–49 years	31,476 (23.0)	28,354 (23.2)	3122 (21.7)	
Maternal education				< 0.001
No education	45,750 (33.5)	40,877 (33.4)	4874 (33.9)	
Primary	42,928 (31.4)	38,756 (31.7)	4172 (29.0)	
Secondary	39,992 (29.3)	35,629 (29.1)	4363 (33.3)	
Higher	8,029 (5.8)	7051 (5.8)	978 (6.8)	
Maternal marital status				0.212
Never married	6,530 (4.8)	5826 (4.8)	704 (4.9)	
Married	123,035 (90.0)	110,047 (90.0)	12,988 (90.3)	
Divorced/widowed/separated	7,141 (5.2)	6442 (5.3)	699 (4.9)	
Maternal employment				< 0.001
Not employed	56,486 (41.3)	49,546 (40.5)	6940 (48.2)	
Employed	80,194 (58.7)	72,748 (59.5)	7446 (51.8)	
Number of under-5 children				0.656
No Under 5	1303 (1.0)	1178 (1.0)	125 (0.9)	
1–3 children	121,426 (88.8)	108,622 (88.8)	12,804 (89.0)	
4 or more children	13,977 (10.2)	12,516 (10.2)	1461 (10.2)	
Wealth index				0.002
Poorest	30,506 (22.3)	27,087 (22.1)	3420 (23.8)	
Poorer	29,057 (21.3)	25,929 (21.2)	3127 (21.7)	
Middle	27,761 (20.3)	24,985 (20.4)	2777 (19.3)	
Richer	26,495 (19.4)	23,793 (19.5)	2702 (18.8)	
Richest	22,885 (16.7)	20,522 (16.8)	2364 (16.4)	
Maternal health behaviour				<0 .001
First quantile (least)	47,853 (35.9)	42,831 (36.0)	5021 (35.7)	
Second quantile	44,560 (33.5)	39,822 (33.4)	4738 (33.7)	
Third quantile	24,083 (18.1)	21,272 (17.9)	2811 (20.0)	
Fourth quantile	6321 (4.8)	5637 (4.7)	683 (4.9)	
Fifth quantile (highest)	10,402 (7.8)	9585 (8.1)	816 (5.8)	
Community-level poverty	62,336 (45.6)	55,652 (45.5)	6683 (46.4)	0.143
Community-level rurality	4141 (3.0)	3682 (3.0)	460 (3.2)	0.295
Community-level illiteracy	59,423 (43.5)	52,966 (43.3)	6458 (44.9)	0.015
Community-level unemployment	63,988 (46.8)	56,931 (46.5)	7057 (49.0)	< 0.001
Community-level unsafe sanitation	64,374 (47.1)	57,494 (47.0)	6880 (47.8)	0.224
Community-level unimproved water source	60,140 (44.0)	53,818 (44.0)	6323 (43.9)	0.927
Community-level limited access to health facility	66,110 (48.4)	59,067 (48.3)	7043 (48.9)	0.313
Human development index				< 0.001
Low HDI	87,712 (64.2)	79,827 (65.3)	7885 (54.8)	
Medium HDI	42,175 (30.9)	36,785 (30.1)	5390 (37.5)	
High HDI	6819 (5.0)	5703 (4.7)	1116 (7.8)	
Cereal yield (kg per hectare)				< 0.001
Tertile 1 (low)	56,808 (41.6)	51,994 (42.5)	4814 (33.5)	
Tertile 2	39,053 (28.6)	35,196 (28.8)	3857 (26.8)	
Tertile 3 (high)	40,845 (29.9)	35,126 (28.7)	5719 (39.7)	
Total fertility rate				< 0.001
Low fertility	8123 (5.9)	6918 (5.7)	1205 (8.4)	
Ideal fertility	6386 (4.7)	5821 (4.8)	565 (3.9)	
High fertility	122,197 (89.4)	109,577 (89.6)	12,621 (87.7)	

### Fixed effects (measures of association)

In Table 3, the results of the five models constructed are shown. With models 1 being an empty model; 2, 3, and 4 showing individual-level, neighborhood-level and country-level variables only respectively; and model 5- the fully adjusted model controlling for the effects of all variables: individual, neighbourhood, and country-level variables.

**Table 3:** Individual, neighborhood and country-level factors associated with the double burden of childhood malnutrition identified by multilevel logistic regression models

	Model 1	Model 2	Model 3	Model 4	Model 5
		OR (95% Crl)	OR (95% Crl)	OR (95% Crl)	OR (95% Crl)
Fixed effect model					
*Individual level factors*					
Age of child (months)		0.98*** [0.98, 0.98]			0.98*** [0.98,0.98]
*Sex (male as ref)*					
Female		0.80*** [0.77, 0.83]			0.80*** [0.77, 0.83]
*Child birth type (single birth as ref)*					
Multiple Births		1.28*** [1.15, 1.42]			1.28*** [1.15, 1.42]
*Diarrhea in the last 2 weeks preceding the survey (No diarrhea as ref)*					
Had Diarrhea		1.06* [1.01, 1.12]			1.06* [1.01, 1.12]
*Child Fever in the last 2 weeks preceding the survey (No fever as ref)*					
Had Fever		1.08** [1.03, 1.13]			1.08** [1.03, 1.13]
*Currently breastfeeding (no as ref)*					
Yes		1.22*** [1.15, 1.29]			1.21*** [1.15, 1.29]
*Maternal age (15–24 years as ref)*					
25–34 years		1.03 [0.98, 1.07]			1.02 [0.98, 1.07]
35–49 years		1.02 [0.96, 1.08]			1.02 [0.97, 1.08]
*Maternal education (no education as ref)*					
Primary education		0.90*** [0.85, 0.95]			0.92** [0.87, 0.98]
Secondary education		0.88*** [0.82, 0.93]			0.89*** [0.84, 0.95]
Higher education		0.94 [0.85, 1.03]			0.95 [0.87, 1.05]
*Maternal marital status (never married as ref)*					
Married or in a union		0.97 [0.88, 1.06]			0.96 [0.88, 1.06]
Divorced/widowed/separated		1.08 [0.95, 1.22]			1.08 [0.95, 1.22]
*Maternal employment (not employed as reference)*					
Employed		0.90*** [0.86,0.94]			0.92*** [0.88, 0.96]
*Number of under-5 children (no under 5 as ref)*					
1–3 children		1.31* [1.04,1.65]			1.29** [1.07,1.56]
4 or more children		1.34* [1.06,1.70]			1.32** [1.08,1.61]
*Wealth index (poorest as ref)*					
Poorer		0.93* [0.88, 0.99]			0.91** [0.86, 0.97]
Middle		0.89*** [0.84, 0.94]			0.86*** [0.80, 0.91]
Richer		0.92* [0.87, 0.99]			0.88*** [0.82, 0.95]
Richest		0.97 [0.90, 1.05]			0.92 [0.85, 1.01]
*Maternal health behavior (first quantile (least) as ref)*					
Second quantile		0.91*** [0.87, 0.96]			0.92** [0.87, 0.97]
Third quantile		0.87*** [0.82, 0.93]			0.88*** [0.83, 0.94]
Fourth quantile		0.97 [0.87, 1.09]			0.97 [0.87, 1.09]
Fifth quantile (highest)		0.93 [0.86, 1.02]			0.94 [0.86, 1.02]
*Neighborhood-level factors*					
Community-level poverty			0.99 [0.94, 1.04]		0.92** [0.86, 0.97]
Community-level rurality			1 [0.86, 1.16]		0.96 [0.82, 1.12]
Community-level illiteracy			1.13*** [1.08, 1.19]		1.10*** [1.04, 1.16]
Community-level unemployment			1.11*** [1.06, 1.16]		1.06* [1.01, 1.11]
Community-level unsafe sanitation			1 [0.96, 1.05]		0.95* [0.91, 1.00]
Community-level unimproved water source			0.97 [0.92, 1.02]		0.96 [0.91, 1.01]
Community-level limited access to health facility			1.04 [0.99, 1.09]		1.04 [0.99, 1.09]
Country-level factors					
*Human development index (low HDI as ref)*					
Medium HDI				1.23 [0.82, 1.84]	1.34 [0.87, 2.06]
High HDI				2.32 [0.99, 5.47]	2.42* [1.04, 5.60]
*Cereal yield (kg per hectare) (tertile 1 (low) as ref)*					
Tertile 2				1.21 [0.82, 1.77]	1.13 [0.68, 1.89]
Tertile 3 (high)				1.46 [0.99, 2.15]	1.47 [0.89, 2.43]
*Total fertility rate (low fertility rate as ref)*					
Ideal fertility rate				1.14 [0.55, 2.37]	1.15 [0.53, 2.51]
High fertility rate				1.58 [0.82, 3.05]	1.43 [0.71, 2.90]
Random effect model					
*Country-level*					
Variance (95% CrI)	1.24 [1.09, 1.42]	1.29 [1.11, 1.51]	1.26 [1.10, 1.44]	1.18 [1.06, 1.31]	1.20 [1.05, 1.37]
VPC (%, 95% CrI)	21.0 [19.1, 23.1]	21.5 [19.3, 24.1]	21.2 [19.2, 23.4]	20.2 [18.6, 21.7]	20.3 [18.4, 22.3]
MOR (95% CrI)	2.89 [2.71, 3.12]	2.95 [2.73,3.23]	2.92 [2.72, 3.14]	2.82 [2.67, 2.98]	2.84 [2.66, 3.05]
*Neighborhood-level*					
Variance (95% CrI)	1.38 [1.33, 1.43]	1.42 [1.36, 1.47]	1.38 [1.33, 1.43]	1.38 [1.34, 1.43]	1.41 [1.36, 1.47]
VPC (%, 95% CrI)	44.3 [42.4, 46.4]	45.1 [42.9, 47.5]	44.5 [42.5,46.6]	43.7 [42.2, 45.4]	44.2 [42.3, 46.3]
MOR (95% CrI)	3.07 [3.00, 3.13]	3.12 [3.04, 3.18]	3.07 [3.00, 3.13]	3.07 [3.02, 3.13]	3.10 [3.04, 3.18]
*Model fit statistics*					
DIC	90,183	84,196	90,150	90,184	84,188
*Sample size*					
Country-level	27	27	27	27	27
Neighborhood-level	13,788	13,485	13,788	13,788	13,485
Individual-level	138,782	132,787	138,782	138,782	132,787

Exponentiated coefficients; 95% confidence intervals in brackets

*p < 0.05, **p < 0.01, ***p < 0.001

Model 1—empty null model, baseline model without any explanatory variables (unconditional model); Model 2—adjusted for only individual-level factors; Model 3—adjusted for only neighbourhood-level factors; Model 4—adjusted for only country-level factors; Model 5—adjusted for individual-, neighbourhood-, and country-level factors (full model); OR—odds ratio, CrI—credible interval, MOR—median odds ratio, VPC—variance partition coefficient, and DIC—Bayesian deviance information criteria

For every one month’s increase in the child’s age, the odd of double burden of malnutrition reduces by 2% (OR: 0.98; 95% CrI 0.98 to 0.98); and 20% reduced odds (OR: 0.80; 95% CrI 0.77 to 0.83) among females compared to males. Children who had diarrhea and fever in the last two weeks preceding the survey had a 6% (OR: 1.06; 95% CrI 1.01 to 1.12) and 8% % (OR: 1.08; 95% CrI 1.03 to 1.13) increase in the odds of double burden of malnutrition respectively. In addition, children of multiple births had a 28% (OR: 1.28; 95% CrI 1.15 to 1.42) increase in the odds of double burden of malnutrition, similar to the 21% (OR: 1.21; 95% CrI 1.15 to 1.29) increase in odds of being malnourished among children who were currently being breastfed as at the time of the survey. Only mothers with primary 8% (OR: 0.92; 95% CrI 0.87 to 0.98) and secondary 11% (OR: 0.89; 95% CrI 0.84 to 0.95) education had decreased odds of having double burden of malnutrition. The odds of double burden of malnutrition increased with the number of under-5 children a mother had, with mothers having between 1 to 3 under-5 children and 4 and more under five children having an increased odd of malnourished children at 29% (OR: 1.29; 95% CrI 1.07 to 1.56) and 32% (OR: 1.32; 95% CrI 1.08 to 1.61) respectively. Conversely, there was an 8% reduction in the odds of malnutrition among mothers in employment (OR: 0.92; 95% CrI 0.88 to 0.96).

Compared to mother-children pairs resident in the poorest households, the odds of double burden of malnutrition decreased across three wealth index categories: poorer wealth index households 9% (OR: 0.91; 95% CrI 0.86–0.97), middle wealth index households 14% (OR: 0.86; 95% CrI 0.80–0.91) and richer wealth index households 12% (OR: 0.88; 95% CrI 0.82–0.95). There was no statistically significant reduction in odds among pairs resident in the richest wealth index households. Similarly, only mothers in the second 8% (OR: 0.92; 95% CrI 0.87–0.97) and third 12% (OR: 0.88; 95% CrI 0.83–0.94) maternal health behavior quantiles had decreased odds of malnutrition compared with mothers in the first (poorest) maternal health behavior quantile. There was no statistically significant reduction in odds among mothers in the fourth and fifth (highest) quantiles of maternal health behavior.

Mother-children pairs living in neighborhoods with a higher prevalence of illiteracy and unemployment had 10% (OR: 1.10; 95% CrI 1.04–1.16) and 6% (OR: 1.06; 95% CrI 1.01–1.11) increased odds of malnutrition respectively. Interestingly, those living in areas with high prevalence of poverty 8% (OR: 0.92; 95% CrI 0.86–0.97) and unimproved sanitation 5% (OR: 0.95; 95% CrI 0.91–1.00) had reduced odds of malnutrition. Pairs resident in countries with high HDI also had a 42% increase in odds of malnutrition (OR: 2.42; 95% CrI 1.04–5.60).

### Random effects (measures of variation)

Table 3 shows the measures of variation from the analysis. We observed a significant variation in odds of malnutrition across neighborhoods (σ2 = 1.38, 95% CrI 1.33–1.43) and countries (*σ*2 = 1.24, 95% CrI 1.09–1.42) in the empty model. The intra-neighbourhood and intra-country correlation coefficient estimated using the intercept component variance shows that 44.3% and 21.0% of variance in odds of malnutrition are consequent upon neighborhood and country level factors respectively. The importance of neighborhood and country contextual factors in malnutrition outcomes is further highlighted by the median odds ratio in Table 3. This is evidenced by the median odds ratio being 3.07 (95% CrI 3.00–3.13) and 2.89 (95% CrI 2.71–3.12) for neighborhoods and countries respectively in the empty model. Furthermore, our all-inclusive model 5 estimates that the odds of a child experiencing malnutrition increases by 3.10-fold (95% CrI 3.04–3.18) and 2.84-fold (95% CrI 2.66–3.05) when the mother–child pair moves to a neighborhood or country with a higher probability of malnutrition respectively.

## Discussion

The results of our analysis show variation in childhood malnutrition across the 27 countries with a low of 6.5% in Burundi to a high of 29.5% in Timor Leste. After adjusting for all level factors, we found that children who had an episode of diarrhea or fever in the last two weeks preceding the survey, were part of multiple birth, currently being breastfed were more likely to have double burden of malnutrition. In addition, mothers of children with more than one under 5-child, were resident in neighborhoods with high illiteracy and unemployment rates and were from countries with high HDI were more likely to have double burden of malnutrition children. Contrastingly, increasing age of child and being of the female sex reduced the likelihood of being malnourished. Similarly, mother of children with some form of education, were employed, improved health behavior and were relatively resident in well off households were less likely to have malnourished children. Curiously, neighborhoods with high levels of poverty and unimproved sanitation were less likely to have double burden of malnutrition children.

Previous work on the double burden of malnutrition had found reduced odds of childhood malnutrition among female children similar to our findings [28]. This can be seen from the significantly higher prevalence of malnutrition among male children in our study. [29] ‘s finding on females being associated with increased odds differ from both ours and [28] ‘s. In contrast to our finding that every one month’s increase in the child’s age reduces the odds of malnutrition, [28] and [29] found that older ages were associated with increased risk of malnutrition. The reduction in odds with age in our study can be explained in the degree of vulnerability to nutritional deficiencies among much younger children [30]. Our findings with respect to maternal education concur with those of [28, 31], and [32]. The protective effect of maternal education, employment and improved health behavior can explained by possible better access to information, knowledge, healthcare, resources and control of decision making which are key in improving nutritional status of children [33, 34].

[32] ‘s finding around the double burden of childhood malnutrition being most common in households with larger number of people can be likened to the positive association of number of under-5 children and multiple births to childhood malnutrition in our analysis. The findings around number of under-5 children as well as multiple births can be situated in the expected reduction in time spent per child and by extension care. Moreover, our study found respondents who reported currently being breastfed were more likely to be malnourished which could be due to children in our study being from age 0–5 years and the recommendation for complementary feeding after the first six months of life. Consequently, dietary adequacy at ages beyond 6 months of age is no longer wholly dependent on breastfeeding, but on complementary feeding which is dependent on feeding practices and dietary quality required to fill the nutritional gap [35, 36].

Previous studies have had similar findings on relatively well-off groups being strong risk factors for the double burden of malnutrition, similar to the community-level poverty and country HDI results of our study [28, 29, 37]. However, this was unlike the reduction in the odds of malnutrition with improvements in the household wealth index category. This divergent finding around income and wealth has been noted in [31, 38] in their study on the double burden of malnutrition among women of child-bearing age in sub-Saharan Africa specifically found wealth as a risk factor for overweight/obesity. Our findings around neighborhood poverty level and HDI although unexpected, follows contrasting findings on socioeconomic status, wealth and income quantiles of previous studies as highlighted in a previous study [31]. Two studies [39, 40] have noted the non-linear effect of country level development assessed using the HDI composite of income, education and life expectancy on individual health outcomes. Moreover, the composite HDI indicator does not adequately capture wide social and health inequalities and complexities [19].

While improved sanitation conditions has been touted in general health and wellbeing, and we found the chances of childhood malnutrition being lower in neighborhoods with unimproved sanitation [36]. The study by [28] on double burden of malnutrition showed no significant relationship and our study finding may be attributed to the noted indirect environmental pathway of sanitation and childhood malnutrition [41]. Our findings on diarrhea and fever in the last two weeks preceding the survey can be explained by malabsorption resulting from repeated diarrhoeal episodes which fever may be a cause or consequence [41].

Evidence of geographical clustering in childhood malnutrition at community and country levels was found in our study with a higher percentage of the variance attributable to neighborhood level factors (44.3%) compared to country level factors (21.0%). In essence, variability consequent upon neighborhood level factors was twice that of country level factors. Merlo et al. [21] have noted the possible similarity in health status of individuals resident in the same geographical areas due to contextual peculiarities. Hence, it would be expected that at both neighborhood and country levels, there would similarities in the prevalence and factors associated with the double burden of childhood malnutrition as a result of proximity, shared experiences and contexts.

A number of our results have also been observed in undernutrition and overnutrition only studies. A pooled analysis of factors associated with undernutrition across 35 low and middle income countries (LMICs) found that maternal education and household wealth index were significant predictors similar to ours [42]. The finding on the negative association of household wealth index have been further buttressed in other multi country studies [43, 44]. Our findings with respect to sex, maternal health behavior, contraction of fever in the two weeks prior to the survey, child birth type, breastfeeding, maternal education, household wealth index, and neighborhood illiteracy are also similar to previous multilevel studies on undernutrition [45–49]. In addition to the above, other studies have also buttressed our findings on the association of age, diarrhea in the two weeks prior to the survey, number of under-5 children, maternal employment and neighborhood unemployment with childhood undernutrition [50–52, 52, 53]. Contrastingly, the risk of undernutrition was found to increase in households with unimproved toilet facility in [54], unlike our finding of reduced risk of childhood malnourishment in neighborhoods with unsafe sanitation.

Studies which have examined childhood overnutrition have also highlighted similar association between maternal education, sex and child malnutrition as our study [55, 56]. However, the association between maternal employment and overweight was found to be non-linear and dependent on the number of hours worked [57, 58]. Two systematic reviews of risk factors of childhood malnutrition have highlighted key predictors of childhood overweight/obesity [57, 58]. With respect to our study results, they found conflicting findings on the effect of socio-economic status on childhood overweight [57, 58]. They also found cessation of breastfeeding or exclusive breastfeeding before four months and childcare attendance as risk factors for childhood overweight [57, 58].

Most of our findings can be situated in the UNICEF Conceptual Framework on the Determinants of Maternal and Child Nutrition which recognizes the multifaceted burden of childhood malnutrition [36]. These determinants classified into immediate, underlying and enabling determinants closely matches our individual, community and country levels factors, validating our multilevel results and highlighting the importance of a wholistic approach targeted at simultaneously reducing the risk of both undernutrition and overweight through tackling the shared drivers. These multilevel interventions must be contextual, evidence driven and systemic taking into consideration geographical clustering of childhood malnutrition evidenced by our results. Furthermore, tackling malnutrition irrespective of type requires a systematic approach that addresses the contributory factors of water, sanitation and hygiene, health, education, adequate maternal nutrition and social protection systems alongside ensuring safe, available, affordable and qualitative diets.

### Study strengths and limitations

This study is an aggregation of nationally representative and generalizable DHS datasets from 27 countries across 5 continents. These are usually high-quality, high response rate datasets from DHS surveys which are conducted using a robust methodology with well documented data sources. These DHS surveys are carried out using standardized survey modules and implementations which allow for comparability across countries. However, these are cross sectional datasets thereby limiting our ability to attribute causality. Moreover, we lacked longitudinal data to assess the length of time respondents have been resident in the neighborhood and exposed to the communal contextual factors to attribute cumulative effects.

## Conclusions

The summary of our analyses shows that children who were wasted/overweight tended to have had an episode of diarrhea or fever in the last two weeks preceding the survey, were part of a multiple birth, were being breastfed at the time of the survey and born to mothers with more than one under 5-child resident in neighborhoods with high illiteracy and unemployment rates. Evidence of geographical clustering in childhood malnutrition at community and country levels was found in our study with variability due to neighborhood level factors twice that of country level factors. Therefore, strategies in tackling the double burden of malnutrition must consider these shared drivers, contextual barriers and geographical clustering effects.

## Supplementary Information

**Additional file 1.** Combined DHS Dataset for 27 Countries.

## Data Availability

All data and datasets generated and/or analysed during the current study are available on the DHS program website https://dhsprogram.com/data (Additional file 1).
